# Comment on ‘Artificial Neural Network Inference Analysis Identified Novel Genes and Gene Interactions Associated With Skeletal Muscle Aging’ by Tarum et al.

**DOI:** 10.1002/jcsm.13680

**Published:** 2024-12-26

**Authors:** Jing‐Lu Zheng, Xi‐Yang Chen, Yi‐Kai Li, Hong‐Wen Liu

**Affiliations:** ^1^ Department of Orthopaedic Zhongshan Hospital of Traditional Chinese Medicine Affiliated to Guangzhou University of Traditional Chinese Medicine Zhongshan China; ^2^ School of Traditional Chinese Medicine Southern Medical University Guangzhou China; ^3^ Clinical Research Center, Department of Orthopaedic Panzhihua Central Hospital Panzhihua China

We read with great interest the recent article by Tarum et al. [1], titled ‘Artificial Neural Network Inference Analysis Identified Novel Genes and Gene Interactions Associated With Skeletal Muscle Aging’. This study introduces an innovative application of artificial neural network inference (ANNi) to elucidate complex gene networks implicated in skeletal muscle ageing. The findings provide significant insights that hold potential for advancing sarcopenia research and guiding targeted interventions. The novel application of ANNi in this context and the compelling results underscore the valuable role computational methods can play in exploring age‐related diseases and identifying new therapeutic targets.

The critical contribution of this study lies in its utilization of ANNi to reveal intricate relationships among genes associated with muscle ageing, specifically identifying CHAD, ZDBF2 and USP54 as central genes. This deep learning–based analysis is precious, as it extends beyond traditional statistical methods to detect subtle gene–gene interactions that may remain hidden in conventional analyses. Through ANNi, the authors ranked genes by their interaction strength, revealing CHAD and ZDBF2 as highly interactive targets within ageing muscle networks, while USP54 emerged as a significant regulator. USP54's role in the ubiquitin–proteasome system, a pathway critical in muscle atrophy, reinforces its relevance as a potential therapeutic target for sarcopenia.

The study's findings provide a more detailed understanding of sarcopenia's molecular landscape. Given the links between age‐related muscle atrophy, heightened catabolic activity, systemic inflammation and oxidative stress, discovering new gene networks provides insights that may eventually inform pharmacological and non‐pharmacological interventions. Tarum et al. effectively demonstrate that ANNi, by examining gene interaction networks rather than focusing solely on differential gene expression, can reveal complex molecular interplay that drives muscle ageing. This perspective allows for a more comprehensive understanding of sarcopenia's pathogenesis, potentially guiding more targeted therapeutic strategies aimed at modulating these interactions to slow or reverse muscle degeneration.

While the study provides valuable insights, there are several areas where additional exploration could further enrich these findings. Although the authors validate gene expression changes through qPCR, assessing how genes like CHAD, ZDBF2 and USP54 express across different stages of sarcopenia would be informative. Understanding whether these genes maintain consistent expression levels throughout muscle ageing or if expression varies across early, mid and late stages could shed light on their roles in sarcopenia progression. Such stage‐based analysis could also reveal time points where therapeutic interventions have maximal impact.

The study also investigates resistance training in older adults, examining its impact on gene expression related to exercise adaptation. However, the finding that specific essential genes did not exhibit differential expression post‐exercise prompts important questions regarding exercise as a regulatory factor in gene expression during ageing. Examining a broader spectrum of exercise types, intensities or durations could provide insights into whether specific training regimens modulate gene interactions differently in ageing muscle [2]. Considering the widely recognized benefits of resistance training in preserving muscle mass among older adults, identifying which regimens optimize gene modulation could be pivotal for designing personalized exercise interventions for sarcopenia prevention.

In addition to exercise, other lifestyle factors, such as nutrition, could interact with gene expression in ways that affect muscle ageing [3]. Exploring the interplay between diet and muscle health, particularly in the context of gene expression, could yield insights that enhance the applicability of these findings to diverse populations. Including dietary considerations in future analyses could offer a more comprehensive understanding of how lifestyle factors might modulate muscle ageing, especially when combined with exercise.

Finally, while ANNi is invaluable for identifying new gene interactions, comparing the predictive power of these genes against established sarcopenia markers, such as Akt, FOXO1 or IL‐6, could clarify their unique contributions [4]. By assessing the roles of CHAD, ZDBF2 and USP54 alongside established markers, it may be possible to refine our understanding of their function within known pathways. Should the newly identified genes demonstrate strong predictive potential, they could serve as supplementary biomarkers, offering a more sensitive means of evaluating sarcopenia onset or progression.

In conclusion, Tarum et al.'s study marks a significant advance in muscle ageing research by demonstrating the potential of artificial intelligence to uncover novel gene networks. This approach enhances our understanding of sarcopenia's molecular complexity and paves the way for future studies focused on therapeutic interventions. We hope that these comments contribute to further explorations in this field, and we look forward to seeing follow‐up studies that build on these valuable findings.

## Conflicts of Interest

The authors declare no conflicts of interest.
